# High-Efficiency Capture of Indole-3-Acetic Acid (IAA) from Water Using AgNPs-Decorated Silicate-Based Nanocomposites

**DOI:** 10.3390/ma19153268

**Published:** 2026-08-02

**Authors:** Rosalia Maria Cigala, Ileana Ielo, Domenico Pio Basile, Francesco Paolo Lamonica, Paola Lanzafame, Georgia Papanikolaou, Giuseppe Zaffino, Francesco Crea, Giovanna De Luca

**Affiliations:** 1 Department of Chemical, Biological, Pharmaceutical and Environmental Sciences, University of Messina, Viale F. Stagno d’Alcontres, 31, 98166 Messina, Italy; domenico.basile@studenti.unime.it (D.P.B.); paola.lanzafame@unime.it (P.L.); georgia.papanikolaou@unime.it (G.P.); francesco.crea@unime.it (F.C.); giovanna.deluca@unime.it (G.D.L.); 2 Ambiente Lab—Technical Services for the Environment and Laboratory Chemical, Biological, Physical, and Environmental Analysis, Nuova Granatari 4, 98164 Messina, Italy; francescopaolo.lamonica@gmail.com (F.P.L.); gzaffino@me.com (G.Z.); 3 National Research Council-Institute for Microelectronics and Microsystems (CNR-IMM), Viale F. Stagno d’Alcontres 31, 98158 Messina, Italy

**Keywords:** auxin, silver nanoparticles, nanocomposites, emerging contaminants, environmental remediation, siliceous materials, clays, diatomaceous earth, adsorption

## Abstract

The ubiquitous use of the plant hormone indole-3-acetic acid (IAA) or auxin in modern agriculture has led to its emergence as a water contaminant, necessitating efficient removal technologies. Addressing the need for high-performance sorbent materials, this study reports the synthesis and characterization of four novel nanocomposites based on halloysite (Hal), bentonite (Ben), sepiolite (Sep), and diatomaceous earth (DE) functionalized with silver nanoparticles (AgNPs). Successful immobilization and morphological features were confirmed via XRD and SEM-EDS. Crucially, post-adsorption EDS analysis provided direct solid-state evidence of pollutant capture through the distinct quantification of organic carbon. High-Performance Liquid Chromatography (HPLC) tests demonstrated that all functionalized materials exhibited a drastically enhanced IAA adsorption capacity over their pristine counterparts during a 96-h kinetic monitoring window. Kinetic profiling revealed a biphasic adsorption pathway characterized by a rapid initial sequestration within the first 10 h followed by a diffusion-limited equilibration, while thermodynamic modeling converged excellently with Langmuir and Sips equations, confirming a surface-confined chemisorption mechanism governed by uniform monolayer deposition. Notably, the performance ranking was found to be primarily governed by the architectural accessibility of the silicate frameworks rather than the absolute magnitude of their specific surface area. Furthermore, solution-phase spectroscopic studies coupled with Dynamic Light Scattering (DLS) and Zeta Potential measurements unraveled a robust, surface-confined ligand exchange mechanism. Rather than triggering colloidal aggregation, IAA coordination induced a controlled, systematic development of an organic molecular shell around the individual silver cores. This work underscores the potential of these engineered AgNPs@silicate platforms as sustainable, high-efficiency materials for the environmental remediation of emerging phytohormone contaminants.

## 1. Introduction

The pervasive application of phytohormones, particularly indole-3-acetic acid (IAA) or Auxin, in modern agriculture and its subsequent runoff have resulted in its classification as an emerging contaminant in aquatic ecosystems [1,2]. Despite the crucial biological role of IAA, its presence in non-target environments poses a potential risk to ecological balance and human health [3,4,5]. Current remediation strategies often fall short in terms of efficiency, selectivity, and cost-effectiveness, highlighting a significant need for innovative materials capable of both efficient pollutant removal and robust structural integrity [6,7,8]. In this context, silicate-based minerals such as clays (bentonite, halloysite, sepiolite) and diatomaceous earth [9,10,11,12,13] have long been recognized as exceptional raw materials for environmental applications due to their high surface area, layered structures, ion-exchange capacity, and abundance [14,15,16]. Furthermore, utilizing these natural clay minerals aligns directly with the core tenets of green chemistry and circular economy principles, as they offer abundant, non-toxic, and highly scalable alternatives to synthetic porous matrices for large scale water remediation [17]. Recent state-of-the-art research has increasingly focused on functionalizing these low-cost supports with metallic nanoparticles to create sophisticated nanocomposites with enhanced catalytic, sensing, and adsorption properties [18,19,20,21]. Specifically, the decoration of clays like bentonite [14,22], halloysite [18,23], and sepiolite [24,25] with AgNPs has emerged as a major focus for environmental applications, and the removal of various pollutants, including organic dyes and heavy metals [24,26]. The well-established function of AgNPs, particularly their broad spectrum antibacterial activity, has historically been the primary driver for their integration into composite materials [27,28]. Furthermore, the colloidal stability of nanoparticles in the presence of natural minerals and other environmental factors is a known challenge, with several studies investigating the phenomena of aggregation and heteroaggregation of AgNPs with clays like kaolin [1,29]. However, despite the extensive literature on AgNPs-silicate nanocomposites, their potential for the adsorption of phytohormone emerging contaminants like IAA [3,30] remains largely unexplored. While AgNPs have been utilized in electrochemical sensors for IAA detection [31,32], this typically involves complex multi-component molecularly imprinted polymers or catalytic amplification, moving away from simple and scalable adsorption-based remediation [33]. Our study directly addresses this gap by creating and comprehensively evaluating a series of four novel nanocomposites: AgNP-functionalized Sepiolite (AgNPs@Sep), Halloysite (AgNPs@Hal), Bentonite (AgNPs@Ben), and Diatomaceous Earth (AgNPs@DE).

The primary objective of this research is to synthesize these four nanocomposites and systematically investigate their performance for enhanced IAA adsorption from aqueous solutions compared to their pristine counterparts. Crucially, this study evaluates the interplay between textural properties and mineral morphology, demonstrating that structural accessibility serves as a more critical determinant for high-efficiency capture than simple surface area extension. A key innovative aspect of this work is the meticulous investigation of the fundamental role of the AgNPs [34]. Unlike prior studies that focus solely on the antibacterial or catalytic roles of AgNPs, we demonstrate how the surface immobilized AgNPs act as adsorption enhancers for IAA, significantly improving the overall removal efficiency of the silicate matrix. In this framework, the surface immobilized AgNPs do not merely act as passive dopants, instead, they serve as specialized chemical anchors that exploit the high coordination affinity of silver facets toward the carboxylate functionalities and the nitrogen atom of the auxin’s indole ring, fundamentally altering the interfacial binding energy of the mineral support [2,35]. The strategic selection of Hal, Ben, Sep, and DE as distinct mineral supports was specifically motivated by their drastically contrasting structural dimensionalities, morphological architectures, and pore geometries. By systematically varying the spatial blueprint of the underlying matrix, spanning from the one-dimensional hollow lumens of halloysite nanotubes and the two-dimensional swellable, stacked clay platelets of bentonite, to the complex three-dimensional fibrous networks of sepiolite and the rigid, open macro-porous biogenic frustules of diatomite, this study also aims to isolate and identify how structural accessibility governs guest-phase immobilization. Rather than merely assessing the absolute specific surface area, this diverse technological screening provides a comprehensive matrix to evaluate how open-channel geometries versus sterically hindered networks optimize the spatial exposure of the active silver sites, thereby dictating both the mass transfer kinetics and the global thermodynamic efficiency of emerging contaminant remediation. We further delve into the mechanistic understanding of this enhancement, utilizing UV-Vis spectroscopy, Dynamic Light Scattering (DLS), and Zeta Potential measurements to monitor the colloidal behavior and stability of AgNPs in the presence of increasing auxin concentration. This multi-analytical approach uncovers a robust, affinity-driven ligand exchange mechanism at the nanoparticle-solvent interface. Rather than triggering conventional colloidal aggregation or structural instability, the incremental coordination of the phytohormone reorganizes the electrical double layer and promotes a controlled, systematic development of an organic molecular shell around the individual metallic cores. This provides crucial molecular level insights into the thermodynamic driving forces that govern the enhanced adsorption subsequently observed on the solid-state composite surfaces, moving the scientific discussion beyond the established antibacterial function of silver nanoparticles [36,37].

Through comprehensive characterization via SEM-EDS, and XRD, coupled with detailed kinetic adsorption studies using HPLC, this work establishes these AgNPs-silicate materials as a potent, multi-functional platform for environmental applications, specifically targeting the removal of emerging phytohormone contaminants.

## 2. Materials and Methods

Distilled water (H_2_O_dist_), absolute Ethanol (EtOH) and Silver acetylacetonate (Ag(acac)) were purchased from VWR International (Radnor, PA, USA). Ultra-pure water, Indole-3-acetic acid (IAA), Halloysite (Hal), Bentonite (Ben), Sepiolite (Sep), and Diatomaceous Earth (DE), were obtained from Sigma-Aldrich (St. Louis, MO, USA). Carbonate-free sodium hydroxide solutions were prepared by diluting concentrated sodium hydroxide ampoules (Riedel-de Haën, Seelze, Germany) and employed as the eluent in HPLC analyses. All chemical reagents were of analytical grade and were used without any further purification.

### 2.1. Synthesis of AgNPs@Silicate-Based Nanocomposites

**Scheme 1:**
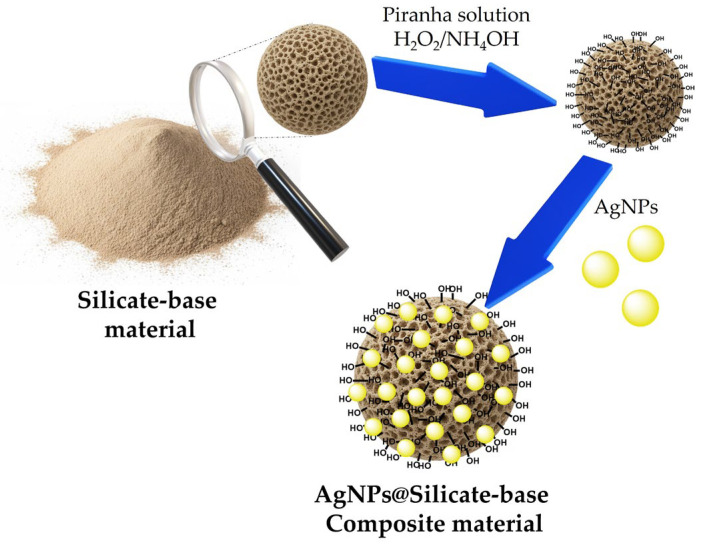
Schematic representation of the two-step synthesis of AgNPs@silicate-based composites.

The AgNPs@silicate-based nanocomposites were synthesized through a two-step process involving the solution-phase synthesis of silver nanoparticles (AgNPs) followed by their subsequent decoration onto the surface of the silicate supports. The green synthesis of the AgNPs was conducted following a methodology adapted from [38,39]. Initially, 4 mg of Ag(acac) were weighed and dissolved in 100 mL of H_2_O_dist_. The solution was stirred on a magnetic plate for 30 min at room temperature. Prior to functionalization, the four silicate/silica materials, Hal, Ben, Sep, and DE, were subjected to a rigorous cleaning/activation procedure [12]. Approximately 200 mg of each silicate material were weighed and washed sequentially: three times with excess H_2_O_dist_ and then three additional times with EtOH. The first wash with EtOH involved suspending the materials in an ultrasonic bath for 30 min. The solvent was then removed, and the materials were oven dried in a forced air oven at 80 °C for 24 h. For the functionalization step, 100 mg of each pre-treated silicate material was individually mixed with 2 mL of the freshly prepared AgNPs solution. The resulting four suspensions were stirred for 5 h at r.t. The stoichiometry was precisely controlled to maintain a molar ratio of Ag/Si = 0.2 for each nanocomposite. It is worth noting, however, that this nominal stoichiometric ratio refers exclusively to the bulk precursor concentrations introduced during the wet synthesis stage; conversely, the local weight and atomic percentages determined via SEM-EDS reflect a surface confined elemental profiling that is inherently lower due to the structural screening of the underlying silicate framework and the localized sampling depth of the electron beam. Following the 5-h reaction, the four resulting suspensions were transferred into 15 mL Falcon tubes and purified by three centrifugation cycles, each performed at 4500 rpm for 15 min. After each cycle, the supernatant was carefully collected and analyzed using UV-Vis spectroscopy. Finally, the recovered functionalized powders (AgNPs@Hal, AgNPs@Ben, AgNPs@Sep, and AgNPs@DE) were dried under vacuum at r.t. for 24 h to yield the final nanocomposite products. The overall multi-step procedure for the preparation of the AgNPs@silicate-based nanocomposites, encompassing the initial green synthesis of silver nanoparticles and their subsequent immobilization onto the cleaned silicate supports, is schematically illustrated in Scheme 1.

### 2.2. UV-Vis Spectroscopy

UV-Vis spectroscopic analyses were conducted using a Stellarnet BLACK-Comet-SR diode array spectrophotometer (Stellarnet mod. SL5, Tampa, FL, USA), equipped with a combined deuterium/tungsten halogen lamp and a multimodal fiber optic cable (length: 1 m; diameter: 1 mm; optical range: 190–1100 nm).

### 2.3. DLS and Zeta Potential Analysis

The Dynamic Light Scattering (DLS) and Zeta Potential (ζ) measurements were conducted at 25 °C utilizing a Malvern Panalytical Zetasizer Nano ZS instrument (Malvern Instruments Ltd. Malvern, UK), equipped with a 4 mW He-Ne laser (λ = 632.8 nm) at a 173° scattering angle. The reported values for the hydrodynamic diameter (D_H_) and electrokinetic potential represent the mean of three independent measurements, with the average absolute deviation cited as the reported uncertainty.

### 2.4. XRD Characterization

XRD analyses were performed using a Bruker D2 Phaser diffractometer (Bruker Corp. Karlsruhe, Germany) equipped with Cu Kα radiation (λ = 1.540598 Å). For all samples, data were collected over a 2θ range of 5–80°, with a step size of 0.025° and an acquisition time of 1 s per step.

### 2.5. SEM-EDS Analysis

The morphological features and elemental composition of the AgNPs@silicate-based nanocomposites were investigated using a FEI Inspect S 50 Scanning Electron Microscope (SEM) (Thermo Fisher Scientific, Waltham, MA, USA), equipped with a tungsten filament source. The analyses were performed in low vacuum modes. The instrument is equipped with Energy Dispersive X-ray Spectroscopy (EDS) detectors, which were utilized for chemical microanalysis, elemental quantification, and surface mapping. Prior to the SEM-EDS microstructural profiling, the nanocomposite powders were mounted onto standard aluminum stubs using double-sided conductive carbon adhesive tape. The specimens were then dried under vacuum and analyzed directly in low-vacuum mode, which naturally mitigates electrostatic charging artifacts on insulating silicate frameworks without requiring prior conductive metal sputtering.

### 2.6. Auxin Quantification by HPLC Analysis

The quantitative determination of residual IAA in the aqueous supernatants was performed using High-Performance Liquid Chromatography (HPLC). The chromatographic system consisted of a DX500 ion chromatographic analyzer (Dionex, Sunnyvale, CA, USA) equipped with a Dionex ED40 amperometric cell, using a pair of glassy carbon and Ag/AgCl electrodes and applying positive potential (0.80 V).

The system was fitted with a guard column (Thermo Scientific Dionex AG16, 4 × 50 mm) and an analytical column (Thermo Scientific Dionex IonPac AS16, 4 × 250 mm). The injection system consisted of a 25 μL loop.

The eluent (30 mM NaOH) was delivered under isocratic conditions at a flow rate of 1.0 mL/min. The system was controlled using Dionex PeakNet 4.10 chromatographic software. Measurements were carried out in temperature-controlled room. The quantification of IAA was achieved through the standard calibration curve (ranging from 1.0 to 30.0 ppm, consistent with the concentrations used in spectroscopic studies). Each sample was analyzed in triplicate to ensure the reproducibility and accuracy of the results, following the experimental protocol described for similar batch systems.

### 2.7. Auxin Adsorption Quantitative Analysis

The adsorption of IAA onto the composite material was investigated in aqueous solution in a batch system. The adsorption experiments were conducted in a 50 mL centrifuge tubes placed in a flat reciprocal shaker (IKA HS 260 basic) at r.t. and 150 rpm. The tubes were kept in the dark throughout the entire process. 15 mg of samples were dispersed in 6 mL of IAA solution (30 mg/L, pH = 4.75) and shaken for a period based on contact time (60, 180, 300, 420, 540, 1440, 2880, 4320, 5760 min). To maintain a rigorously constant solid-to-liquid ratio of 2.5 g/L across all replicates, the exact volume of the IAA solution was continuously adjusted in accordance with the precise analytical mass of the nanocomposite sample. Specifically, while the nominal benchmark setup involved 15 mg of sorbent immersed in 6 mL of solution, any minor deviation in the measured solid mass was immediately compensated for by modifying the corresponding liquid volume, thereby ensuring that the specific boundary conditions and concentration gradients remained perfectly identical and stable throughout the entire kinetic monitoring window. After adsorption, solid and liquid phases were separated by centrifugation at 4500 rpm for 15 min at r.t. Once the phases are separated, the supernatant is filtered using cellulose acetate syringe filters. The residual concentration of IAA in the collected supernatant was quantified via HPLC. The amount of the adsorbed IAA at time t, q_t_ (mg/g), was calculated using the following Equation (1):

(1) $$q_{t}=\frac{\left(C_{0}-C_{t}\right)\cdot V}{m_{ads}}$$

where C_0_ and C_t_ are the initial IAA concentration (in mg/L) and the IAA concentration at time t, respectively, V is the volume of the adsorption solution (mL) and m_ads_ is the mass of adsorbent (g). The amount of IAA adsorbed at equilibrium time was denoted as q_e_. The influence of IAA concentration was investigated for the concentrations of 5, 10, 15, 20, and 30 mg/L at r.t.

All experiments were performed in triplicate, and the reported values represent the mean of these measurements.

### 2.8. Batch Adsorption Kinetics and Modeling

The experimental q_t_ versus time (t) profiles were interpreted through non-linear regression analysis. The experimental datasets for each engineered platform were fitted to the integrated non-linear forms of the Pseudo-First Order (PFO) and Pseudo-Second Order (PSO) kinetic models, expressed by Equations (2) and (3), respectively:

(2) $$q_{t}=q_{e}\left(1-e^{-k_{1}t}\right)$$

where q_t_ and q_e_ (mg/g) are the amounts of IAA adsorbed at time t and at equilibrium, respectively, and k_1_ (min^−1^) is the first-order rate constant.

(3) $$q_{t}=\frac{k_{2}q_{e}^{2}t}{1+k_{2}q_{e}t}$$

where k_2_ (g mg^−1^ min^−1^) is the second-order rate constant. The mathematical convergence, goodness-of-fit, and structural correlation of each model were rigorously cross-examined and evaluated based on the resulting adjusted coefficients of determination (R^2^).

### 2.9. Adsorption Isotherm Models

The equilibrium adsorption isotherms were investigated through a series of batch experiments conducted at room temperature under strict dark conditions. In each experiment, a fixed amount of the adsorbent (15 mg) was thoroughly dispersed in 6 mL of IAA aqueous solutions at varying initial concentrations, specifically 5, 10, 15, 20, and 30 ppm. The mixtures were placed in 50 mL centrifuge tubes and shaken on a flat reciprocal shaker at 150 rpm for a sustained contact period of 48 h. Afterward, the phases were separated through centrifugation process, and the supernatant was filtered through cellulose acetate syringe filters before being analyzed to determine the remaining analyte concentration.

To describe the equilibrium distribution of IAA between the aqueous solution and the nanocomposite surfaces, the experimental data were interpreted using four different isotherm models: Langmuir, Freundlich, Temkin, and Sips. The non-linear forms of these models were applied to determine the characteristic parameters of the adsorption process.

The Langmuir isotherm assumes that adsorption occurs on a homogeneous surface with a finite number of identical active sites, leading to the formation of a single monolayer. Its non-linear expression is given by Equation (4):

(4) $$q_{e}=\frac{q_{max}K_{L}C_{e}}{1+K_{L}C_{e}}$$

where C_e_ (mg/L) is the equilibrium concentration of the analyte, q_max_ (mg/g) represents the maximum monolayer adsorption capacity, and K_L_ (L/mg) is the Langmuir constant related to the affinity of the binding sites.

The Freundlich isotherm is an empirical model used to describe adsorption on heterogeneous surfaces and multilayer adsorption. It is expressed as:

(5) $$q_{e}=K_{F}C_{e}^{1/n}$$

where K_F_ [(mg/g)(L/mg)^1/n^] is the Freundlich constant related to the adsorption capacity and 1/n (dimensionless) is the heterogeneity factor.

The Temkin isotherm explicitly accounts for the interactions between the adsorbent and the adsorbate, assuming that the heat of adsorption decreases linearly with surface coverage. The model is defined as:

(6) $$q_{e}=\frac{RT}{b_{T}}ln(A_{T}C_{e})$$

where A_T_ (L/mg) is the Temkin equilibrium binding constant and b_T_ (kJ/mol) is a constant related to the heat of adsorption.

Finally, the Sips isotherm is a hybrid model that combines the features of the Langmuir and Freundlich equations to better describe heterogeneous adsorption systems. It follows the equation:

(7) $$q_{e}=\frac{q_{max}{\left(K_{s}C_{e}\right)}^{\beta_{s}}}{1+{\left(K_{s}C_{e}\right)}^{\beta_{s}}}$$

where K_s_ (L/mg) is the Sips equilibrium constant, and β_S_ is the Sips heterogeneity exponent. The goodness-of-fit for each model was evaluated based on the coefficient of determination (R^2^).

## 3. Results

**Figure 1:**
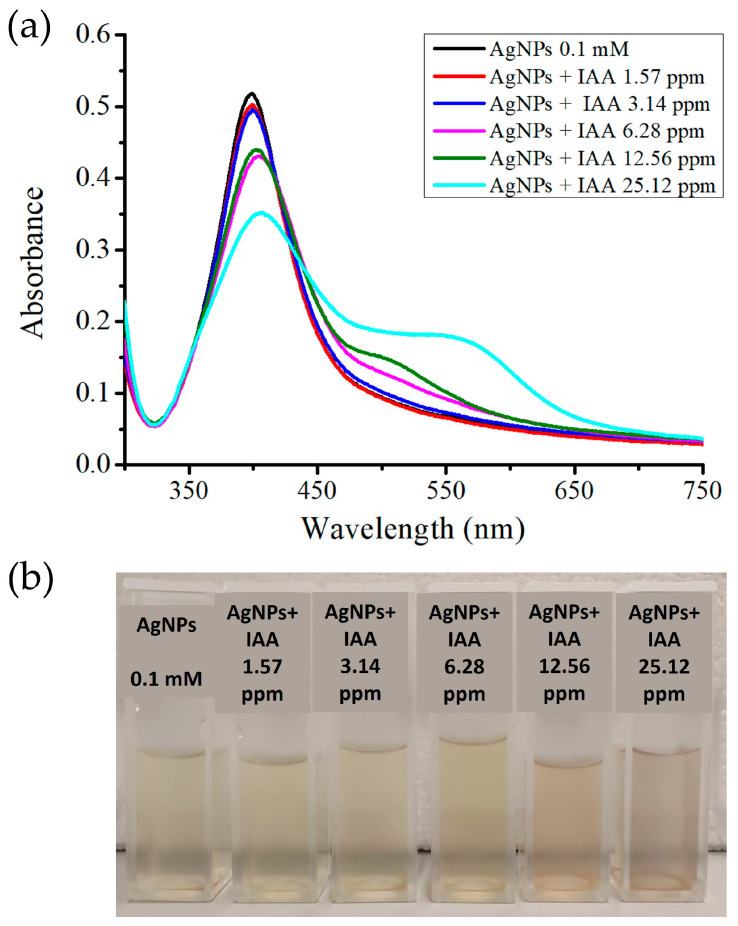
(**a**) UV-Vis absorption spectra of pristine colloidal AgNPs 0.1 mM, black line treated with increasing concentrations of IAA from 1.57 ppm to 25.12 ppm; (**b**) Visual appearance and color evolution of the corresponding AgNPs-IAA colloidal mixtures inside the spectrophotometric cuvettes.

The successful synthesis and subsequent structural, morphological, and interfacial characterization of the AgNPs@silicate nanocomposites were systematically evaluated alongside their performance in IAA remediation. Visually, the reduction of the silver precursor solution was initially signaled by a distinct color transition from colorless to pale yellow, a macroscopic phenomenon directly corroborated by UV-Vis spectroscopy which revealed the characteristic Localized Surface Plasmon Resonance (LSPR) band of colloidal AgNPs at approximately 400 nm (Figure 1a, black-line). To systematically unravel the interfacial affinity between silver and the target phytohormone, the free-colloid AgNPs + IAA system was initially prioritized as an unconstrained solution-phase model. Investigating the nanomaterials in their freely suspended state effectively eliminates the severe spectroscopic background interference, scattering artifacts, and mass-transfer limitations inherently imposed by bulk silicate matrices, thereby allowing high-resolution tracking of the pristine coordination chemistry via liquid-phase characterizations. Crucially, while the immobilization onto diatomite, halloysite, sepiolite, and bentonite structurally restricts the dynamic rotational freedom of the active silver sites, the underlying chemical nature of the Ag-IAA interaction is expected to translate consistently to the hybrid platforms. The localized surface-confined ligand exchange, electrostatic double-layer compression, and organic shell growth observed in the free colloidal models serve as a direct mechanistic blueprint for the behaviour of the anchored silver nanoparticles, defining the primary driving force behind the enhanced macroscopic adsorption capacity of the engineered nanocomposites.

To establish a fundamental chemical and spectroscopic baseline for the heterogeneous system, the solution-phase interaction between the colloidal AgNPs (0.1 mM) and increasing aliquots of the IAA analyte (1.57, 3.14, 6.28, 12.56, and 25.12 ppm) was investigated via incremental UV-Vis analysis, displaying characteristic spectral shifts (Figure 1a,b). Figure 1b provides the corresponding visual profile of the solutions within the cuvettes. The pristine silver nanoparticles (black line) exhibit a sharp, symmetrical LSPR band centered at 398 nm, which is highly characteristic of well-dispersed, spherical AgNPs. As the concentration of IAA increases, a progressive decrease in the intensity of the primary LSPR peak is observed, accompanied by a slight redshift and a broadening of the absorption profile. Crucially, at the highest analyte concentrations (12.56 and 25.12 ppm), a distinct secondary broad absorption band emerges in the 500–600 nm region (green and cyan line). This spectral modification is macroscopically mirrored by the subtle color transition of the colloidal suspension shown in Figure 1b, which shifts from a pale, clear yellow to a deeper, slightly brownish hue at higher IAA loadings. It is highly critical to highlight that this secondary plasmonic feature and the concurrent optical darkening are not triggered by colloidal instability, irreversible particle aggregation, or precipitation. This is macroscopically and unambiguously confirmed by the visual evidence in Figure 1b, where all samples remain perfectly homogeneous and transparent, completely lacking any visible precipitate, turbidity, or sedimentation at the bottom of the cuvettes. Instead, this red-shifted spectral fingerprint represents the distinctive spectroscopic signature of a strong electronic coupling between the indole ring and the carboxylate functionalities of the IAA molecule with the silver surface [32]. The progressive chemisorption of the deprotonated phytohormone severely alters the local refractive index, significantly compressing the electrical double layer and modifying both its effective thickness and dielectric constant. Consequently, the appearance of the band at 560 nm stems from interfacial chemical modifications and local electronic perturbations rather than a bulk physical clustering of the metallic cores, perfectly matching the structural behavior unraveled by the subsequent DLS and ζ investigations.

**Figure 2:**
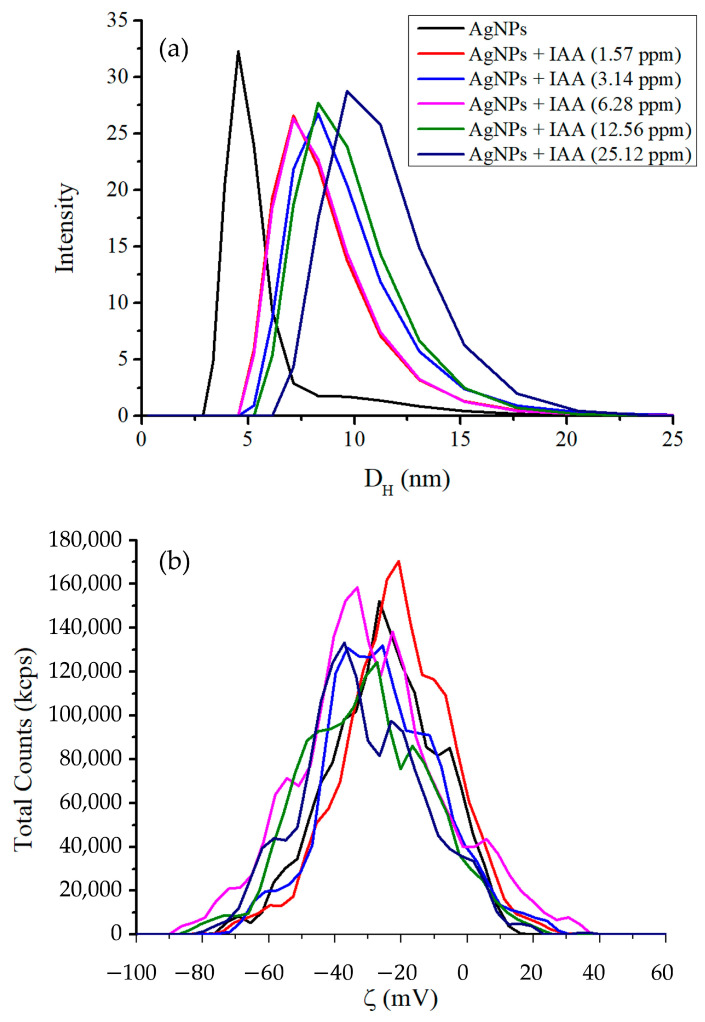
(**a**) DLS intensity distributions displaying the monomodal nanoscale hydrodynamic diameter (D_H_) of pristine colloidal AgNPs (0.1 mM) and its evolution upon incremental IAA additions (1.57–25.12 ppm); (**b**) corresponding ζ distribution curves showing the electrical surface charge modifications as a function of IAA increasing concentration.

To provide a quantitative cross-examination of the interfacial phenomena observed via UV-Vis spectroscopy, DLS size distributions and ζ profiles were systematically recorded (Figure 2a,b). As illustrated in Figure 1a, the pristine AgNPs colloidal suspension (0.1 mM, black line) exhibits a highly uniform, monomodal hydrodynamic size distribution confined entirely within the ultra-small nanoscale domain, featuring a sharp intensity peak centered at a D_H_ of (4.6 ± 0.5) nm. This pristine state is electrokinetically stabilized by a robust negative surface charge, with the ζ profile (Figure 1b) displaying a broad, well-defined distribution peaking at (−24.0 ± 2.0) mV at an initial solution pH of 6.78. This negative ζ guarantees sufficient electrostatic repulsion to maintain a fully dispersed colloidal system, matching the clear, pale yellow optical profile observed in the spectrophotometric cuvettes. Upon the incremental titration of IAA, the colloidal system bypasses conventional, uncontrolled bulk aggregation pathways, which would otherwise trigger an immediate shift towards the micrometer range or massive peak broadening, and instead displays a distinct, step-like interfacial reorganization. Crucially, the D_H_ does not exhibit a continuous, concentration-dependent growth as the phytohormone titration progresses; rather, a prominent and discrete structural transition occurs exclusively between the native AgNPs and the IAA-exposed states. The mere transition from the pristine suspension to the lowest IAA exposure (1.57 ppm) triggers an immediate rearrangement of the capping architecture, shifting the peak D_H_ from the baseline value of 4.6 nm up to the 7.2–8.3 nm range. Throughout the remaining titration matrix, as the IAA concentration escalates up to the maximum loading of 25.12 ppm, the intensity profiles in Figure 2a remain remarkably stable and tightly confined within a strict nanometric envelope, settling at a maximum of (9.6 ± 1.2) nm at the highest concentration (dark blue line). In this regard, the marginal, incremental variations observed in the D_H_ values across the higher concentration range are statistically compensated for by the broadening of the standard deviation, falling strictly within the measurement’s experimental error margin. This behavior provides direct steric evidence that a stable organic molecular coating is fully established at the early stages of interaction, with the primary coordination shell maintaining its structural integrity and thickness regardless of further analyte influx. Concurrently, the electrokinetic environment tracks these structural shifts through an intricate coordination mechanism (Figure 2b). The initial addition of 1.57 ppm IAA promotes a temporary increase in surface negativity down to (−29.6 ± 1.0) mV, indicating the rapid, early-stage anchoring of anionic phytohormone species onto the silver facets. Beyond this threshold, higher IAA exposures shift the ζ distributions towards less negative values, settling at (−17.4 ± 0.9) mV at the maximum 25.12 ppm concentration.

The rapid adsorption process and the associated electrokinetic evolution is accompanied by a pH decrease from 6.78 to 5.76. The interaction with the silver surface is favored by the predominance of the deprotonated IAA^−^ species in the pH range (pKa ≈ 4.75) [40].

The combined DLS and ζ trends confirm a surface-confined ligand exchange mechanism that perfectly clarifies the optical and spectral phenomena observed in the UV-Vis section. The influx of IAA ions triggers a highly competitive displacement process, where the deprotonated phytohormone expels the original, bulkier capping agents from the metallic core, binding tightly to the silver surface via its carboxylate groups or the indole ring nitrogen. The exceptional reduction in hydrodynamic size observed at 6.28 ppm highlights the formation of a highly compressed, tightly bound molecular shell. At the highest analyte concentrations (12.56 and 25.12 ppm), the continuous influx of H^+^ ions induces an electrostatic screening effect that compresses the electrical double layer, reducing the magnitude of the negative ζ without compromising colloidal stability. The comprehensive hydrodynamic diameters, peak electrokinetic potentials, and corresponding solution pH shifts recorded throughout this titration matrix are systematically compiled in Table 1.

The immobilization efficiency of these formed nanoparticles onto the mineral matrices was verified by UV-Vis analysis of the post-centrifugation supernatants collected during the washing cycles, which confirmed a total depletion of residual, non-immobilized AgNPs in the waste solutions (Figure S1), thereby establishing their complete deposition.

**Figure 3:**
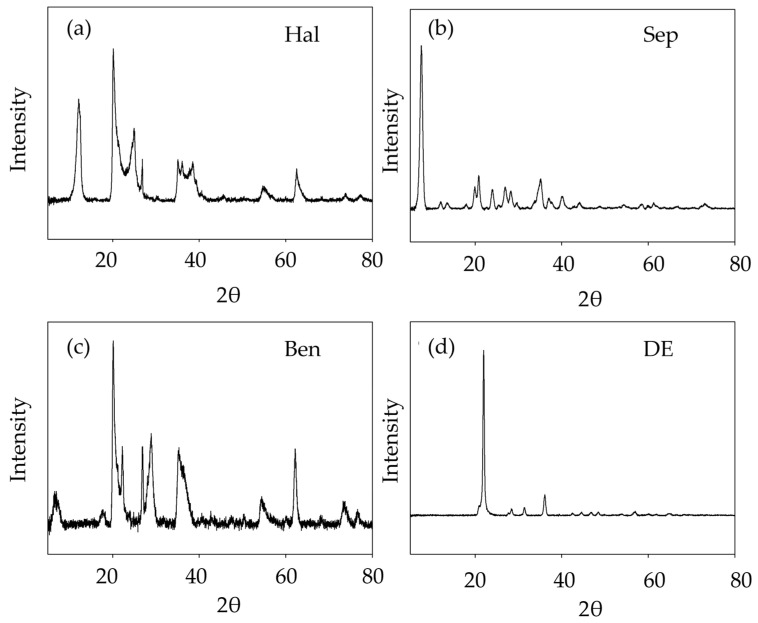
The XRD patterns of (**a**) halloysite, (**b**) sepiolite (**c**) bentonite and (**d**) diatomite.

The structural integrity of silicate support was evaluated via XRD analysis. The patterns for halloysite, sepiolite, bentonite, and diatomite exhibited their typical diffraction peaks, indicating that the functionalization process did not compromise the crystalline framework of the minerals. Figure 3a shows the typical diffraction peaks of halloysite, observed at 2θ values of 12.0°, 20.1°, 24.6°, 35.0°, 37.9°, 54.5°, and 62.6°, which correspond to the (001), (100), (002), (110), (003), (210), and (300) planes, respectively [41]. The XRD pattern for sepiolite, illustrated in Figure 3b, is characterized by its most prominent reflection at a low angle of 2θ = 7.0°, which corresponds to the (001) plane and reveals a basal spacing (d_001_) of 1.26 nm. Further diagnostic peaks are identified at 2θ values of approximately 11.8°, 20.5°, 23.5°, 26.5°, and 35.0°, which match the standard patterns for natural sepiolite crystals [42]. Figure 3c illustrates the diffraction pattern of bentonite, characterized by peaks at 2θ = 20.7°, 26.5°, 36.3°, and 54.7°, associated with the (110), (210), (124), and (144) planes, respectively [9]. Finally, the XRD pattern of diatomite shown in Figure 3d exhibits a prominent peak at 2θ = 21.9°, corresponding to the (101) plane of cristobalite, along with additional peaks at 28.4°, 31.4°, and 36°, assigned to the (111), (102), and (200) planes, respectively [43].

**Figure 4:**
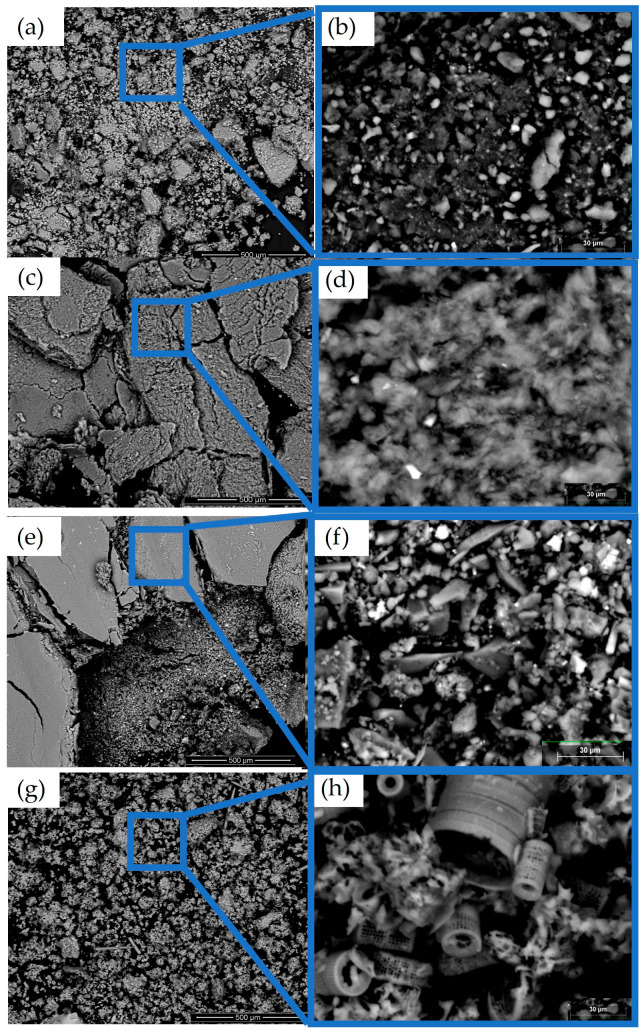
SEM micrographs showing the nanocomposites at dual magnifications: (**a**,**b**) AgNPs@Hal; (**c**,**d**) AgNPs@Sep; (**e**,**f**) AgNPs@Ben; and (**g**,**h**) AgNPs@DE. Panels (**a**,**c**,**e**,**g**) illustrate a wide-field panoramic view (scale bar = 500 μm); Panels (**b**,**d**,**f**,**h**) display the corresponding high-magnification regions of interest (scale bar = 30 μm; accelerating voltage of 25 kV).

The surface morphology and elemental distribution of the AgNPs@silicate nanocomposites were investigated via SEM and EDS to confirm successful functionalization and provide direct evidence of IAA capture. The morphological features and surface topography of the nanocomposites were thoroughly cross-examined at dual scales, as depicted in Figure 4. At low magnification (Figure 4a,c,e,g, scale bar = 500 μm), the panoramic micrographs reveal distinct texturing and grain size distributions characteristic of each mineral matrix. Pristine-like structural integrity is preserved across all samples post-functionalization, confirming that the green synthesis protocol does not cause alterations or chemical degradation of the underlying mineral architectures. High-magnification insights (Figure 4b,d,f,h, scale bar = 30 μm) unwrap the unique micro-morphological traits that govern the surface accessibility of the material series. For AgNPs@Hal (Figure 4b) and AgNPs@Ben (Figure 4f), the surfaces are dominated by typical clustered clay domains and rigid, irregularly broken aluminosilicate platelets, respectively. In contrast, AgNPs@Sep (Figure 4d) exhibits a highly dense, dense-packed network of intertwined fibrous bundles. Crucially, AgNPs@DE (Figure 4h) stands out by showcasing perfectly preserved, highly symmetric biogenic silica frustules. The distinct cylinder-like structures and open macro-porous channels of the DE remain completely unblocked after AgNPs immobilization. This open-network architecture explains the outstanding performance observed in mass-transfer dynamics, as it provides a geometrically optimized, three-dimensional platform that maximizes the exposure and spatial accessibility of the active silver anchor sites.

**Figure 5:**
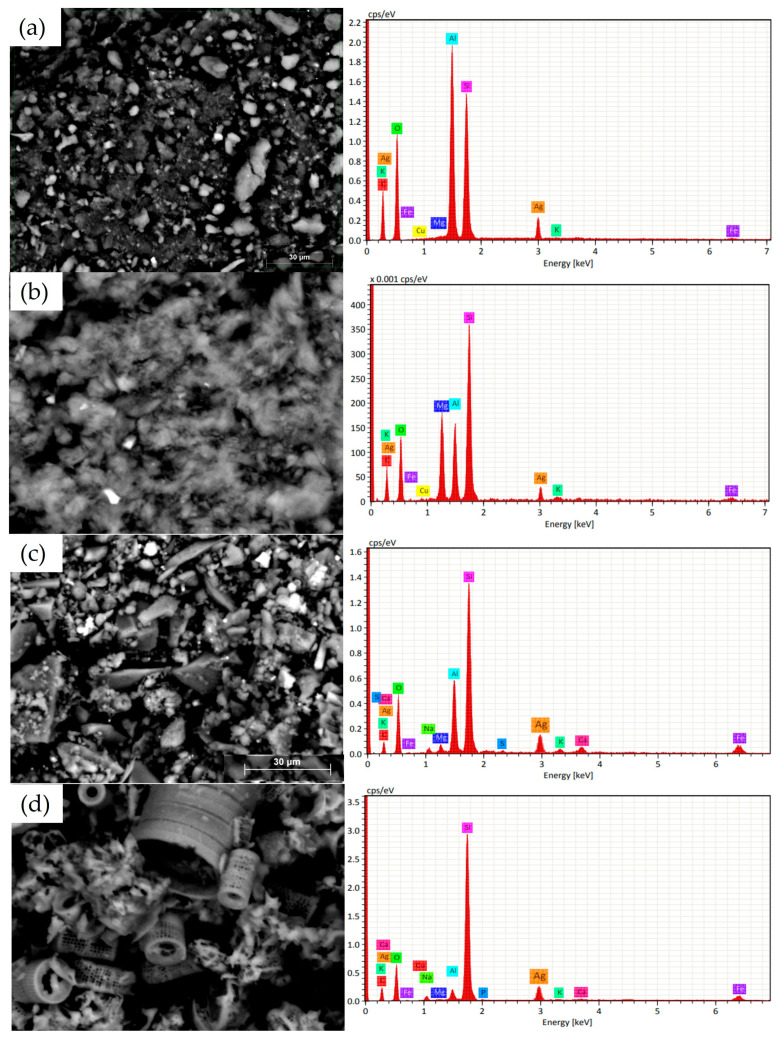
EDS elemental mapping of silver recorded at 200× magnification for the nanocomposites: (**a**) AgNPs@Hal, (**b**) AgNPs@Sep, (**c**) AgNPs@Ben, and (**d**) AgNPs@DE.

AgNPs@Hal and AgNPs@Ben appear as heterogeneous granular aggregates and fragmented plates, while AgNPs@Sep retains its characteristic fibrous bundle structure. AgNPs@DE is dominated by the biogenic, highly porous skeletal remains typical of diatomaceous earth. To evaluate the spatial distribution and chemical homogeneity of the metallic phase post-functionalization, EDS elemental mapping focusing on the Ag signal was systematically performed at a baseline magnification of 200×, as displayed in Figure 5.

The elemental maps unwrap a dense, regular network of luminescent points, providing clear visual evidence of a highly homogeneous distribution of AgNPs across all four matrices, regardless of their radically different mineral architectures. Crucially, the complete absence of isolated macro-clusters, significant metallic blank zones, or irregular phase segregation indicates that the green synthesis protocol effectively mitigates nanoparticle sintering and uncontrolled aggregation during the loading phase. This uniform surface decoration is a vital structural asset; ensuring that the active silver anchor sites are evenly dispersed across the available basal planes and open channels prevent localized site saturation and maximizes the effective interfacial area available for dynamic coordination with the target organic molecules.

Quantitative elemental composition of the functionalized platforms before and after the 48-h batch remediation experiments was determined via EDS analysis, as summarized in Table 2.

The successful immobilization of AgNPs onto the different crystalline lattices is confirmed by the net detection of elemental silver in all pristine composites, yielding mass percentages (% mass Ag) of 0.08% for AgNPs@Hal, 0.04% for AgNPs@Sep, 0.15% for AgNPs@DE, and peaking at 1.13% in the case of AgNPs@Ben. Concurrently, the structural framework of the underlying mineral matrices is reflected by the significant silicon mass fractions (% mass Si), which range consistently from 19.76% up to 36.91% depending on the specific silicate or carbonaceous-silicate structure. Crucially, a clear compositional distinction emerges when evaluating the carbon content (% mass C) across the two operational stages. In their pre-adsorption states, all four hybrid nanocomposites display a total absence of elemental carbon (0%), confirming the high chemical purity of the initial activated supports and the complete removal of organic synthesis residuals during the ultrasonic washing steps. Following the 48-h incubation period with the phytohormone solution, a dramatic, unambiguous appearance of carbon signals is recorded across all matrices. Specifically, the carbon content jumps sharply to 5.26% for AgNPs@Sep, 7.86% for AgNPs@Ben, 9.17% for AgNPs@Hal, and reaches a maximum of 9.45% for AgNPs@DE. This systematic accumulation of organic carbon provides direct, solid-state chemical proof of successful IAA capture, showcasing a strong correlation with the macro-scale equilibrium loadings unraveled by the isotherm modeling.

**Figure 6:**
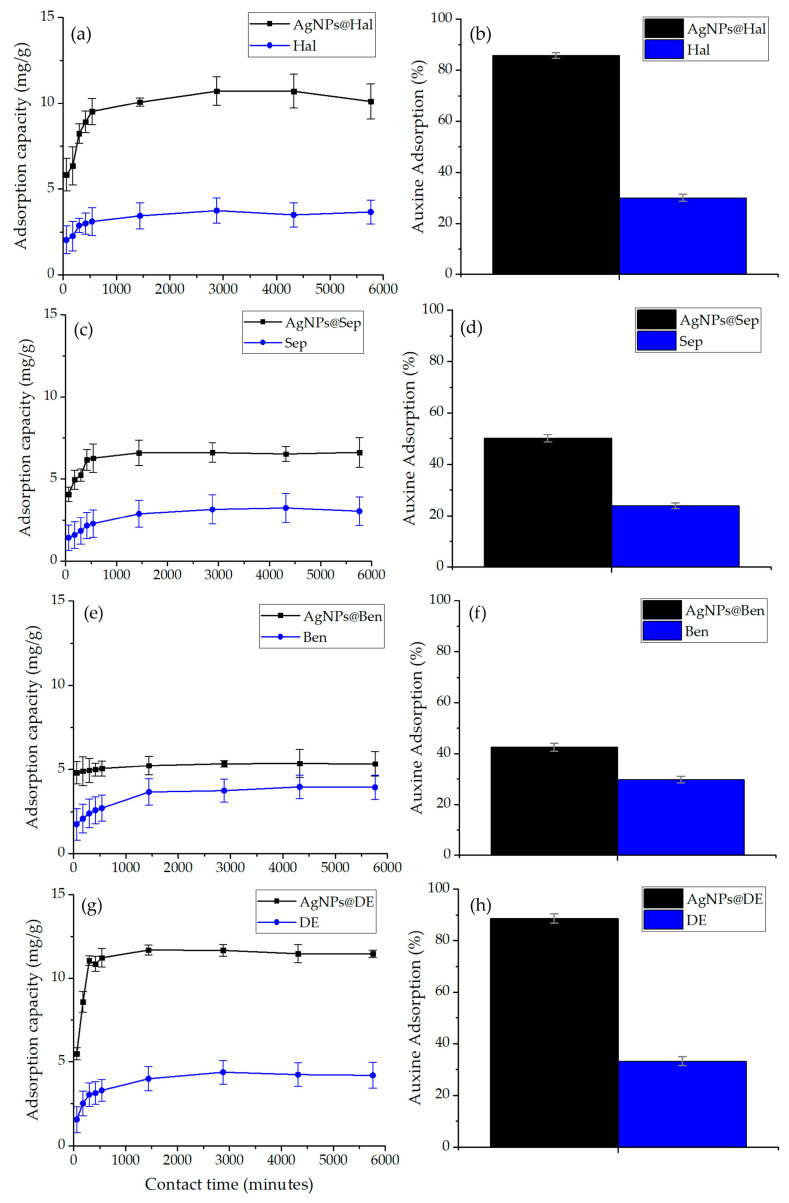
Time-dependent batch adsorption profiles and corresponding removal efficiencies for IAA remediation onto raw and AgNPs-decorated silicate matrices: (**a**,**b**) AgNPs@Hal vs. pristine Hal; (**c**,**d**) AgNPs@Sep vs. pristine Sep; (**e**,**f**) AgNPs@Ben vs. pristine Ben; (**g**,**h**) AgNPs@DE vs. pristine DE. Left panels represent the kinetic evolution of the experimental loading capacity (q_e_ mg/g) over a 96-h contact time matrix; right panels display the comparative final auxin adsorption efficiency percentages attained at equilibrium (48 h). (Adsorption conditions: 15 mg of adsorbent, 6 mL of IAA solution 30 mg/L, 25 °C, in the dark).

The macroscopic performance of the primary silicate frameworks and their corresponding AgNPs@silicate was systematically cross-examined through time-dependent batch uptake profiles and total removal efficiency percentages (Figure 6).

To gain deep molecular-level insights into the mass transfer dynamics and determine the rate-controlling step governing the phytohormone uptake, the experimental time-dependent data were systematically modeled using the non-linear formulations of the Pseudo-First Order (PFO) and Pseudo-Second Order (PSO) kinetic equations. The calculated kinetic parameters, including the theoretical equilibrium loading capacities (q_e_, calc), specific rate constants (k_1_ and k_2_), and their respective coefficients of determination (R^2^), are compiled in Table 3. A comparative cross-examination of the statistical parameters uncovers a clear bifurcation between the two theoretical frameworks. The PFO model demonstrates an insufficient performance in simulating the global adsorption process; this is mathematically evidenced by noticeably low R^2^ value and, most importantly, a severe underestimation of the calculated q_e_ when mirrored against the real experimental endpoints (q_e_, exp). Such a mismatch indicates that the rate-determining step cannot be ascribed to a simple, physisorption-dominated process governed by a mere concentration gradient at the boundary layer. Conversely, the experimental kinetic profiles converge with outstanding mathematical precision toward the non-linear PSO model. All four functionalized hybrid platforms exhibit an excellent agreement between the experimentally recorded uptakes q_e_, exp and the corresponding mathematically derived capacities q_e_, calc. This statistical excellence rigorously confirms that the rate-determining step is governed by a surface-confined chemisorption mechanism. In this interfacial framework, the rate of sequestration is directly driven by the formation of strong, shared-electron coordinating interactions between the surface-active silver facets and the specific chemical anchors of the auxin molecule (the deprotonated carboxylate group and the nitrogen heteroatom of the indole ring), leading to a highly regularized monolayer configuration. Beyond the maximum thermodynamic capacities, the PSO rate constants (k_2_) unravel the crucial role played by the underlying mineral architecture in dictating the physical accessibility of the active sites. Beyond the maximum thermodynamic capacities, the parameters derived from the non-linear PSO model require careful interpretation. Interestingly, the calculated rate constants (k_2_) compiled in Table 3 display higher absolute values for the lower-performing platforms (AgNPs@Ben and AgNPs@Sep) compared to the high-capacity nanocomposites (AgNPs@DE and AgNPs@Hal). This behavior represents a well-documented mathematical artifact of the non-linear PSO hyperbolic function; for matrices with restricted equilibrium uptakes (q_e_), the fitting algorithm artificially forces a higher k_2_ value to accommodate the sharp, immediate transition toward a low-lying plateau [44]. Conversely, from a physical and experimental standpoint, the true kinetic remediation velocity defined as the real-time mass of pollutant captured per unit of time during the initial operating window is dramatically optimized in the order of AgNPs@DE > AgNPs@Hal > AgNPs@Sep > AgNPs@Ben. This enhanced mass transfer is directly enabled by the open, unhindered geometric blueprints of biogenic diatomite and hollow halloysite nanotubes, which grant the fluid phase rapid and unhindered intraparticle diffusion access to the active silver anchor sites, whereas the stacked layers of bentonite impose severe steric and spatial limitations.

As illustrated in the kinetic curves (Figure 6a,c,e,g), all investigated materials display typical biphasic adsorption behavior. This is characterized by an initial, rapid uptake phase during the first 10 h of interaction, driven by the high availability of vacant active sites, followed by a slower, asymptotic approach toward a steady-state equilibrium plateau fully established between 48 and 96 h. Crucially, the surface decoration with AgNPs yields a dramatic, long-range enhancement in the loading capacities (q_e_) across all four mineral configurations, expanding the thermodynamic boundaries of the raw matrices without modifying the overall kinetic profile shapes. A detailed analysis of the individual mineral frameworks uncovers specific structural influences on IAA capture. The DE platform exhibits the highest absolute improvement and the absolute best performances among the series. Specifically, the functionalized AgNPs@DE matrix (Figure 6g) achieves a maximum experimental loading capacity (q_e_) of nearly 11.5 mg/g compared to the restricted ~4.4 mg/g of untreated DE. This exceptional performance is corroborated by the corresponding end-point removal analysis (Figure 6h), where the AgNPs embedding triggers a massive efficiency increase, jumping from a modest 33% (raw mineral) to a prominent 89% removal rate for the hybrid composite. Similarly, the tubular clay hybrid AgNPs@Hal (Figure 6a) demonstrates strong remediation capabilities, reaching a loading capacity close to 10.7 mg/g against the 3.8 mg/g limit of pristine Hal, effectively boosting the total auxin extraction percentage from 30% to an optimal 86% (Figure 6b). Conversely, the fibrous and platy minerals exhibit slightly lower overall performance metrics, though the relative enhancement induced by the silver decoration remains highly significant. The sepiolite-based hybrid AgNPs@Sep (Figure 6c) reaches an equilibrium loading capacity of approximately 6.6 mg/g relative to the ~3 mg/g recorded for raw Sep, driving the total removal efficiency from 23% up to 50% (Figure 6d). Lastly, the bentonite system exhibits the most conservative uptake variations; the functionalized AgNPs@Ben complex (Figure 6e) stabilizes at a maximum q_e_ of roughly 5.4 mg/g compared to the 3.8 mg/g displayed by pristine Ben. This trend is mirrored in Figure 6f, where the total auxin removal registers a controlled, stepwise shift from 30% (raw matrix) to 43% (nanocomposite). This clear disparity in the maximum loading enhancements ranking in the order of AgNPs@DE > AgNPs@Hal > AgNPs@Sep > AgNPs@Ben, strongly reflects the specific macrostructural properties of the underlying supports. The rigid, highly open macro-porous architecture of the diatomite frustules and the easily accessible lumen of the halloysite nanotubes provide a significantly more effective and unhindered mass transfer of the phytohormone compared to the densely packed interstratified layers of bentonite or the tightly bound crystalline fiber bundles of sepiolite. This structural advantage maximizes the spatial exposure and accessibility of the highly active silver binding sites, optimizing the solid-state trapping efficiency.

**Figure 7:**
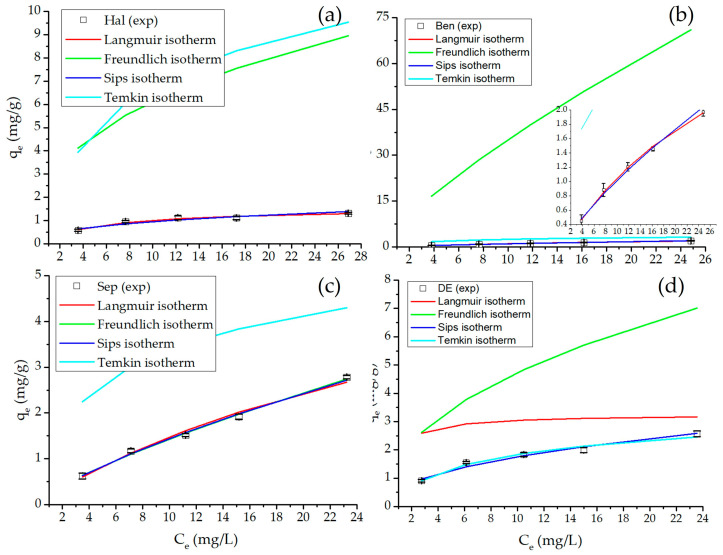
Non-linear equilibrium adsorption isotherms of IAA onto the pristine, untreated silicate-based mineral supports: (**a**) bare halloysite, (**b**) bare bentonite, with inset showing the magnified low-scale experimental range), (**c**) bare sepiolite, and (**d**) bare diatomaceous earth. Experimental data points (black squares) are fitted with Langmuir (red lines), Freundlich (green lines), Sips (blue lines), and Temkin (cyan lines) isotherm models. (Adsorption conditions: 15 mg of adsorbent, 6 mL of IAA solution, 25 °C, in the dark). The vertical lines extending from the experimental data points (squares) represent the error bars associated with each measure.

To deeply evaluate the surface affinity and baseline adsorption mechanisms of the untreated mineral supports prior to nanoparticle decoration, the equilibrium isotherms of pristine halloysite, bentonite, sepiolite, and diatomaceous earth were systematically investigated. The experimental datasets, plotting equilibrium loading capacity (q_e_ mg/g) against equilibrium concentration (C_e_ mg/L), were fitted using non-linear Langmuir, Freundlich, Sips, and Temkin mathematical models, as illustrated in Figure 7. At a macroscopic level, all four pristine silicates exhibit drastically low baseline adsorption capacities toward IAA within the evaluated concentration range, with maximum experimental q_e_ values strictly remaining below 3.0 mg/g. Specifically, pristine bentonite (Figure 7b) shows the lowest performance, barely reaching an experimental capacity of ~2.0 mg/g at the highest concentration (magnified in the inset plot), while halloysite (Figure 7a) exhibits an even lower uptake, flattening out at around 1.3 mg/g. Pristine sepiolite (Figure 7c) and diatomaceous earth (Figure 7d) demonstrate slightly higher baseline captures, positioning their maximum experimental points at approximately 2.8 mg/g and 2.6 mg/g, respectively. From a modeling perspective, the non-linear regression curves unwrap a critical discrepancy among the four theoretical models depending on the specific mineral matrix. For Hal and Ben, both the Freundlich (green line) and Temkin (cyan line) isotherms completely fail to simulate the experimental behavior, severely overestimating the loading capacities across the entire concentration window. A distinct behavior is observed for pristine Sep (Figure 7c); in this framework, while the Freundlich curve successfully tracks the continuous upward experimental trend, the Temkin model exhibits a complete failure, drifting away from the data points at mid-to-high equilibrium concentrations. Conversely, a unique modeling mismatch emerges for bare DE (Figure 7d). In this case, both the Freundlich and Langmuir equations fail to properly describe the interfacial phenomenon. The Freundlich model significantly overestimates the target uptake, whereas the classic Langmuir equation assumes an abrupt, premature plateauing behavior above C_e_ = 6 mg/L (q_max_ ~ 3.1 mg/g), completely missing the steady, slight upward trend displayed by the experimental points at higher equilibrium concentrations. For DE, only the Sips and Temkin models successfully converge alongside the experimental dataset, accurately capturing the marginal energetic heterogeneity inherent to the raw biogenic framework.

This massive overestimation is particularly striking for halloysite and bentonite, where the Freundlich and Temkin curves diverge exponentially up to predicted values of 9–10 mg/g and 70 mg/g, respectively, losing physical consistency with the true material performance. Conversely, the Langmuir (red line) and Sips (blue line) models display an excellent, overlapping convergence with the experimental data points (black squares) for Hal, Ben, and Sep, tracking the linear to plateau transition accurately and suggesting a highly uniform, monolayer-driven positioning on a finite number of active sites. A minor deviation in model behavior is uniquely observed for pristine diatomaceous earth (Figure 7d). In this case, while the Freundlich model still significantly overestimates the target capacities, the Temkin model converges tightly alongside the Sips and Langmuir models. For DE, the Langmuir equation assumes a strict, classic plateauing behavior above C_e_ = 6 mg/L (q_max_ ≈ 3.1 mg/g), whereas the Sips and Temkin models closely follow the continuous, slight upward trend of the experimental points at higher equilibrium concentrations. This behavior indicates that while the raw clays maintain a structurally homogeneous surface topology toward the target phytohormone, the pristine porous frustules of diatomite retain a marginal degree of energetic heterogeneity inherent to their carbonaceous-silicate hybrid matrix.

**Figure 8:**
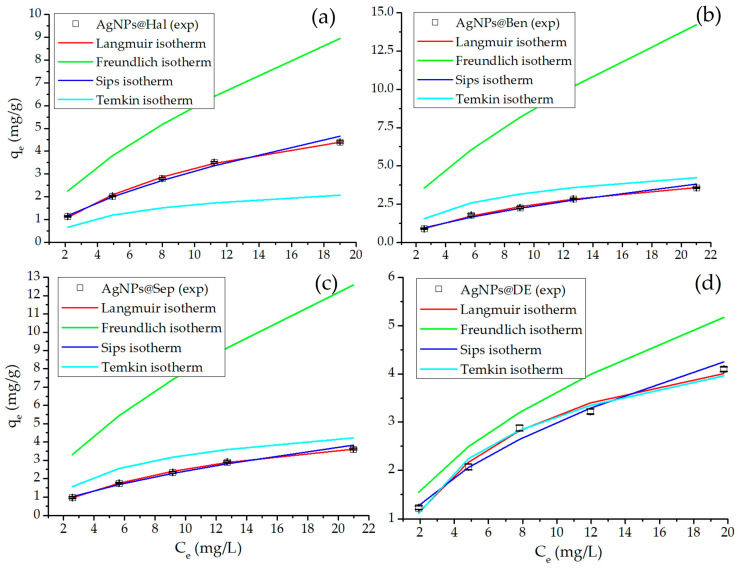
Non-linear equilibrium adsorption isotherms of IAA onto the AgNPs-decorated silicate nanocomposites: (**a**) AgNPs@Hal, (**b**) AgNPs@Ben, (**c**) AgNPs@Sep, and (**d**) AgNPs@DE. Experimental data points (black squares) are fitted with Langmuir (red lines), Freundlich (green lines), Sips (blue lines), and Temkin (cyan lines) isotherm models. (Adsorption conditions: 15 mg of adsorbent, 6 mL of IAA solution, 25 °C, in the dark). The vertical lines extending from the experimental data points (squares) represent the error bars associated with each measure.

Following the baseline evaluation of the raw supports, the equilibrium adsorption isotherms of the corresponding AgNPs-decorated nanocomposites were systematically analyzed to ascertain the thermodynamic impact of the metallic functionalization. The experimental isotherms, fitted via non-linear Langmuir, Freundlich, Sips, and Temkin models, are illustrated in Figure 8. The deployment of four distinct mathematical approximations allows for a comprehensive, multi-perspective interpretation of the surface-analyte interactions. Specifically, while the Langmuir model probes the feasibility of a localized, ideal monolayer deposition on homogeneous sites, the empirical Freundlich equation tests for multilayer accumulation over energetically heterogeneous frameworks. Concurrently, the Temkin isotherm explicitly accounts for indirect sorbent-sorbate interactions by tracking linear variations in the heat of adsorption, whereas the hybrid Sips model evaluates the potential transition from cooperative, multi-site behavior at low concentrations to uniform monolayer saturation at higher equilibrium stages.

Compared to the pristine substrates, the surface decoration with AgNPs induces a distinct and systematic enhancement in the thermodynamic adsorption profiles across the entire equilibrium concentration window. The maximum experimental loading capacities increase significantly for all hybrid frameworks, demonstrating the superior affinity of the newly created silver-based active sites toward the target phytohormone. Specifically, AgNPs@Hal (Figure 8a) reaches an experimental capacity of approximately 4.4 mg/g at C_e_ ≈ 19 mg/L, while both AgNPs@Ben (Figure 8b) and AgNPs@Sep (Figure 8c) track a closely matched equilibrium trajectory, stabilizing at maximum experimental points of ~3.6 mg/g at C_e_ ≈ 21 mg/L. The highest performance is consistently maintained by the diatomaceous earth composite, AgNPs@DE (Figure 8d), which hits an experimental capacity of 4.1 mg/g at a lower equilibrium concentration (C_e_ ≈ 19.8 mg/L), confirming a steeper initial slope and a more energetic surface-analyte interaction. From a mathematical modeling perspective, the introduction of AgNPs modifies the fitting convergence of the theoretical models, though some critical discrepancies remain. The Freundlich isotherm (green line) continuously overestimates the loading performance across all four functionalized systems, failing to grasp the classic saturation trend of the surface-active sites. Similarly, the Temkin model (cyan line) demonstrates a poor correlation for AgNPs@Hal, AgNPs@Ben, and AgNPs@Sep, severely underestimating the uptake capacities at mid-to-high equilibrium concentrations, though it shows a markedly improved convergence for AgNPs@DE (Figure 8d). Crucially, the non-linear Langmuir (red line) and Sips (blue line) equations provide the most accurate and statistically consistent description of the experimental data points (black squares) for all composites. For AgNPs@Ben and AgNPs@Sep, the Langmuir and Sips curves overlap almost perfectly, accurately simulating a homogeneous, monolayer-driven chemisorption mechanism onto the uniform binding arrays created by the immobilized AgNPs. Interestingly, for AgNPs@Hal and AgNPs@DE, a slight divergence between the two models becomes visible at higher equilibrium concentrations. In both cases, the Sips model tracks the experimental points with excellent precision, showing a marginal upward curvature compared to the strict flattening of the Langmuir plateau. This subtle variation indicates that while the high-affinity AgNPs sites govern the primary monolayer anchoring, the overall adsorption process preserves a minor degree of structural and energetic heterogeneity that is deeply inherent to the underlying silicate-carbonaceous hybrid frameworks.

To quantitatively consolidate the thermodynamic insights extracted from the non-linear regression curves, the calculated numerical parameters for the Langmuir, Freundlich, Temkin, and Sips models are systematically summarized in Table 4.

A cross-examination of the thermodynamic parameters reveals that for the AgNPs@DE composite, the kinetic equilibrium capacity (q_e_ ~ 11.5 mg/g in Table 3) stands noticeably higher than the maximum monolayer saturation capacity derived from the isotherms (q_max_ ~ 5.5 mg/g in Table 4). This phenomenon could be ascribed to the unique mass transfer hydrodynamics governing the biogenic, open macro-porous architecture of the diatomite frustules. During the extended 96-h kinetic monitoring window, the continuous, long-term mechanical stirring maximizes fluid-phase penetration into the deepest internal porous channels of the silicate framework. The high initial concentration gradient (30 mg/L) could act as a strong driving force that overcomes intraparticle diffusion resistance, driving the IAA anions to successfully access and saturate highly active, deeply embedded silver anchor sites that are only partially reached during the standard isotherm equilibrium profiling. This progressive chemical scaling highlights that the unhindered geometry of the diatomite scaffold could allow the hybrid platform to maximize its localized chemisorption potential over time, approaching the absolute mass balance boundary.

A detailed mathematical cross-examination of the correlation coefficients (R^2^) rigorously confirms that the surface functionalization with AgNPs alters the underlying adsorption mechanism, inducing an excellent shift toward surface homogeneity and monolayer-controlled uptake. For the pristine mineral matrices, the Langmuir and Sips equations yield low maximum theoretical monolayer capacities, validating the macroscopic observations. Bare Hal displays the lowest affinity framework with a q_max_ of 1.5630 mg/g, followed by pristine DE at 3.2605 mg/g and raw Ben at 4.6860 mg/g. Bare Sep exhibits a higher calculated q_max_ value of 6.8728 mg/g, but its extremely low Langmuir affinity constant (K_L_ = 0.0274 L/mg) combined with a poor determination coefficient (R^2^ = 0.897) indicates that the active sites on the raw clay are highly scattered and thermodynamically unfavorable for coordinated IAA binding. Upon decoration with AgNPs, the thermodynamic profile experiences a profound optimization. The maximum adsorption capacity of AgNPs@Hal undergoes a striking four-fold increase, rising from 1.5630 mg/g to 7.1685 mg/g, backed by an outstanding statistical fit (R^2^ = 0.9947). Similarly, AgNPs@Ben experiences a capacity expansion up to 6.0132 mg/g with a jump in the Langmuir correlation coefficient from 0.9777 to 0.9945. In the case of AgNPs@DE, the theoretical monolayer capacity increases substantially to 5.5218 mg/g (R^2^ = 0.9871). For the AgNPs@Sep, while the calculated q_max_ shifts to 5.9347 mg/g, the key modification lies in the dramatic regularization of its interactions; the Langmuir fit jumps from the poor baseline value of 0.897 up to an excellent R^2^ = 0.9978, and its affinity constant increases nearly three-fold (K_L_ = 0.0740 L/mg). This clear trend mathematically demonstrates that the immobilized silver nanoparticles successfully mask the native, irregular adsorption environments of the clay, replacing them with a highly dense and energetically uniform array of silver-anchored active sites. Further confirmation of this interfacial reorganization is provided by the Freundlich heterogeneity factor (1/n) and the Sips heterogeneity exponent (β_S_). For the functionalized composites, the values of 1/n settle consistently within the 0.5184–0.6572 range, indicating a highly favorable chemisorption process. Concurrently, the Sips exponent increases significantly post-functionalization, for instance, shifting from 0.3954 to 0.656 for halloysite, moving closer to unity. Because a β_S_ value of 1 denotes a perfectly homogeneous Langmuir system, this systematic upward shift confirms that the immobilization of AgNPs creates a more uniform, monolayer-like energetic landscape. Finally, the Temkin heat of adsorption parameters (b_T_) show a consistent drop for Ben, DE, and Sep post-decoration, chemically reflecting a more balanced, unhindered exothermic interaction between the deprotonated phytohormone (IAA^−^) and the silver-decorated interfaces, concluding that these engineered hybrid platforms are highly optimized thermodynamic sinks for target water remediation.

## 4. Discussion

The findings of this research confirm the initial hypothesis that surface-immobilized AgNPs serve as primary adsorption enhancers rather than mere antibacterial dopants. The dramatic increase in loading capacity, specifically the four-fold enhancement for halloysite and the transition of diatomaceous earth to a 70% removal rate, demonstrates that the silver decoration successfully reorganizes the mineral interface. This interfacial reorganization effectively masks the native, thermodynamically unfavorable attracting environments of the raw silicates and replaces them with a dense, uniform array of high-affinity silver facets. The adsorption process onto the AgNPs-decorated silicates follows a biphasic kinetic pathway, as evidenced by the time-dependent uptake profiles. This behavior is characterized by a rapid initial sequestration of IAA during the first 10 h, facilitated by the high density of accessible silver anchor sites, followed by a slower, diffusion-limited approach to thermodynamic equilibrium between 48 and 96 h. The high statistical correlation with the Langmuir and Sips models (R^2^ > 0.99) suggests that the rate-determining step is a surface-confined chemisorption mechanism. In this framework, the deprotonated IAA^−^ anions undergo a ligand exchange process to attract tightly to the silver facets, replacing the native, irregular adsorption environments of the raw minerals with a uniform and energetically favorable monolayer.

The mechanistic insights derived from the interfacial analysis are crucial for interpreting this performance. The observed surface confined ligand exchange indicates that the deprotonated phytohormone IAA^−^ displaces original capping agents to anchor tightly to the silver surface through its carboxylate groups or indole ring nitrogen.

This strong, affinity-driven interaction explains the excellent fit of the Langmuir and Sips models, which mathematically confirm a transition toward surface homogeneity and monolayer-controlled uptake post-functionalization. These results move the scientific discussion of nanocomposites beyond established antibacterial functions toward a specialized role in high-selectivity environmental remediation.

The experimental adsorption capacities observed for the synthesized nanocomposites can be further contextualized by evaluating the specific surface area (SSA) of the raw materials and their functionalized counterparts. Raw halloysite displays a characteristic SSA of approximately 57.3 m^2^/g [45], while bentonite and sepiolite typically exhibit significantly distinct baseline values, spanning from 39.0 to 77.0 m^2^/g for pristine bentonite clay varieties and reaching as high as 130 m^2^/g for highly fibrous sepiolite matrices [46,47]. In contrast, raw diatomaceous earth generally presents the lowest baseline SSA, reported between 17.4 and 27.8 m^2^/g [48].

Upon functionalization with AgNPs, the surface parameters undergo distinct modifications depending on the silicate matrix. For instance, AgNPs-modified bentonite has been shown to reach an SSA of 94.48 m^2^/g [49], while in the case of diatomite, the impregnation of AgNPs into the purified framework resulted in a surface area of 39 m^2^/g [50]. Interestingly, the adsorption efficiency ranking established in this work, AgNPs@DE > AgNPs@Hal > AgNPs@Sep > AgNPs@Ben, does not directly correlate with the absolute magnitude of the total surface area. Although it is not possible to establish a direct correlation between the SSA and the adsorption capacity, the obtained results suggest that the porous architecture of the material and the accessibility of the active sites can influence the IAA uptake to a greater extent than the extension of the available surface area alone. Specifically, the open macro porous channels of the diatomite frustules and the accessible lumens of the halloysite nanotubes facilitate unhindered mass transfer, ensuring that the target molecules can effectively reach the highly active silver anchor sites. Conversely, the more densely packed interstratified layers of bentonite and the fiber bundles of sepiolite likely present greater steric hindrance, limiting the overall trapping efficiency despite their high theoretical surface areas. As summarized in Table 5, the specific surface area measurements provide a quantitative basis for comparing the textural properties of the investigated supports.

Furthermore, the comparative performance of the four supports underscores the critical role of mineral architecture in pollutant capture. The superior efficacy of AgNPs@DE and AgNPs@Hal compared to bentonite and sepiolite can be attributed to the open macro-porous architecture of the diatomite frustules and the accessible lumens of the halloysite nanotubes, which facilitate unhindered mass transfer of the IAA molecules. This suggests that the geometric accessibility of the support is just as vital as the chemical affinity of the functionalizing nanoparticles for optimizing solid-state trapping.

To fully unravel the structural factors driving the remediation efficiency, a deep correlation must be established between the morphological pore networks of the hybrid matrices and their macroscopic performance. Crucially, the experimental data demonstrates that the magnitude of the SSA is not the primary descriptor governing IAA capture. While Sep displays the highest baseline surface area due to its dense, crystalline fibrous bundles, it underperforms significantly when compared to the diatomite and halloysite frameworks. This apparent paradox highlighted the vital role played by the internal porosity and geometric architecture of the nanocomposites. The rigid, biogenic macro-porous channels of diatomite and the wide, open hollow lumens of halloysite nanotubes provide an ideal, sterically unhindered microenvironment. This open porosity ensures two distinct advantages: first, it allows for a highly dispersed, uncoagulated anchoring of the AgNPs guest phase during the wet-chemical functionalization; second, it minimizes intraparticle diffusion resistance during the fluid-phase batch runs. The target IAA anions can rapidly diffuse through these open channels, fully accessing the high-density array of active silver anchor sites embedded deep within the porous matrix. On the contrary, the highly tortuous, micro-porous channels of sepiolite and the narrow, swellable interlayer spaces of the stacked bentonite platelets impose severe spatial constraints and steric hindrance. This structural confinement triggers localized pore-blocking effects post-decoration and macroscopically translates into restricted thermodynamic capacities and a diffusion-limited kinetic pathway, proving that open-pore architectural accessibility outweighs mere high-SSA baselines in emerging phytohormone sequestration.

In a broader context, these AgNPs@silicate platforms offer a scalable and high-efficiency solution for the removal of emerging agricultural contaminants like IAA, which pose significant risks to aquatic ecosystems. Future research directions should prioritize the evaluation of these materials in complex, real-world water matrices to investigate the effects of competing organic pollutants. Additionally, exploring the regeneration potential of the silver-decorated surfaces will be essential for developing sustainable, long-term remediation technologies for diverse phytohormone contaminants.

## 5. Conclusions

In this study, a series of four novel hybrid nanocomposites (AgNPs@Hal, AgNPs@Sep, AgNPs@Ben, and AgNPs@DE) were successfully engineered via a green synthesis protocol and evaluated for IAA phytohormone remediation from aqueous systems. Macro-scale batch experiments unraveled a dramatic enhancement in IAA capture driven by the immobilized metallic phase, following a biphasic kinetic pathway that reached equilibrium within 48–96 h. Among the series, AgNPs@DE and AgNPs@Hal demonstrated outstanding remediation performances, yielding maximum experimental loading capacities of approximately 11.5 mg/g and 10.7 mg/g, respectively, which correspond to prominent endpoint removal efficiencies of up to 70–85%. Crucially, these outcomes demonstrate that pollutant capture efficiency is primarily dictated by the architectural accessibility of the open-pore silicate frameworks rather than the absolute magnitude of their specific surface area. Rather than triggering bulk colloidal aggregation, the incremental influx of the deprotonated phytohormone promoted a systematic, controlled growth of a surface-confined organic molecular shell around the individual metallic cores (with the hydrodynamic diameter increasing from 4.6 to 9.6 nm). Non-linear modeling and solid-state EDS analysis consolidated these insights, proving that the surface-immobilized AgNPs successfully masked the native, irregular binding environments of the raw silicates, replacing them with a uniform, high-affinity array of active silver anchors governed by a monolayer chemisorption mechanism. In conclusion, this work establishes these sustainable AgNPs@silicate materials as potent, structurally robust platforms for environmental remediation. Furthermore, by intercepting a target molecule that acts as a dangerous gut-derived uremic toxin, these hybrid matrices open up new technological avenues for water purification in regions suffering from heavy bacteriological compromises, where the intrinsic biocide nature of silver can be paired with high-capacity adsorption to eliminate dual biological and chemical threats. Future research directions will prioritize evaluating these platforms within complex, real-world wastewater matrices to assess the impact of competing species, alongside deep-diving into long-term recyclability and fixed-bed column scaling for advanced water treatment.

## Figures and Tables

**Table 1:** Hydrodynamic diameter (D_H_), Zeta Potential, and pH values of colloidal AgNPs upon incremental IAA addition.

Sample	Peak D_H_ (nm)	Peak Zeta Potential (mV)	pH
AgNPs 0.1 mM	4.6 ± 0.5 *	−24.0 ± 2.0	6.78
AgNPs + IAA 1.57 ppm	7.2 ± 0.6	−29.6 ± 1.0	6.45
AgNPs + IAA 3.14 ppm	8.3 ± 0.8	−27.4 ± 1.0	6.31
AgNPs + IAA 6.28 ppm	7.2 ± 0.7	−25.6 ± 3.0	6.09
AgNPs + IAA 12.56 ppm	8.3 ± 0.9	−17.7 ± 1.0	5.84
AgNPs + IAA 25.12 ppm	9.6 ± 1.2	−17.4 ± 0.9	5.76

* The standard deviation associated with measurements represents the average of three independent replicates.

**Table 2:** EDS elemental composition (mass percentage) of the AgNPs@silicate nanocomposites before and after the 48-h IAA adsorption process.

Sample	% Mass Ag (Pre-Adsorption)	% Mass Si (Pre-Adsorption)	% Mass C (Pre-Adsorption)	% Mass C (Post-Adsorption)
AgNPs@Hal	0.08	19.76	0	9.17
AgNPs@Sep	0.04	25.14	0	5.26
AgNPs@Ben	1.13	29.44	0	7.86
AgNPs@DE	0.15	36.91	0	9.45

**Table 3:** Kinetic parameters for IAA adsorption onto AgNPs-decorated silicate nanocomposites.

**Sample**	**q_e_ exp (mg/g)**	PFO			PSO		
		**q_e_ calc (mg/g)**	k_1_ (min^−1^)	R^2^	q_e_ calc (mg/g)	k_2_ (g mg^−1^ min^−1^)	R^2^
AgNPs@Hal	10.7 ± 0.8	10.0 ± 0.5	(7.4 ± 1.7) × 10^−3^	0.64	10.5 ± 0.3	(1.3 ± 0.3) × 10^−3^	0.87
AgNPs@Sep	6.6 ± 0.6	6.3 ± 0.2	(1.3 ± 0.3) × 10^−2^	0.63	6.6 ± 0.1	(3.2 ± 0.6) × 10^−3^	0.92
AgNPs@Ben	5.4 ± 0.2	5.2 ± 0.1	(4.5 ± 1.0) × 10^−2^	0.18	5.2 ± 0.1	(2.8 ± 0.9) × 10^−2^	0.86
AgNPs@DE	11.5 ± 0.4	11.4 ± 0.2	(9.2 ± 0.8) × 10^−3^	0.96	11.7 ± 0.2	(1.4 ± 0.2) × 10^−3^	0.93
Hal	3.8 ± 0.7	3.4 ± 0.2	(8.2 ± 2.1) × 10^−3^	0.58	3.6 ± 0.1	(4.0 ± 0.92) × 10^−3^	0.87
Sep	3.2 ± 0.9	3.0 ± 0.2	(3.5 ± 0.8) × 10^−3^	0.72	3.2 ± 0.2	(1.9 ± 0.5) × 10^−3^	0.86
Ben	3.8 ± 0.7	3.7 ± 0.2	(3.6 ± 0.8) × 10^−3^	0.69	3.9 ± 0.2	(1.6 ± 0.4) × 10^−3^	0.86
**DE**	4.4 ± 0.7	4.1 ± 0.2	(4.5 ± 0.7) × 10^−3^	0.87	4.3 ± 0.1	(1.7 ± 0.2) × 10^−3^	0.98

**Table 4:** Mathematical parameters calculated from the Langmuir, Freundlich, Temkin, and Sips isotherm models for IAA adsorption onto pristine mineral supports and their respective AgNPs@silicate nanocomposites.

Sample	Isotherm			
	Langmuir	Freundlich	Temkin	Sips
Hal	q_max_ = 1.5630 mg/g	K_F_ = 2.5378 (mg/g)(L/mg)^1/n^	A_T_ = 1.1684 L/mg	β_S_ = 0.3954
	K_L_ = 0.1812 L/mg	1/n = 0.383	b_T_ = 0.8954 kJ/mol	K_S_ = 1.98 × 10^−5^ L/mg
	R^2^ = 0.989	R^2^ = 0.9107	R^2^ = 0.9498	R^2^ = 0.9123
AgNPs@Hal	q_max_ = 7.1685 mg/g	K_F_ = 1.3817 (mg/g)(L/mg)^1/n^	A_T_ = 1.2870 L/mg	β_S_ = 0.656
	K_L_ = 0.0833 L/mg	1/n = 0.6343	b_T_ = 3.8258 kJ/mol	K_S_ = 0.00088 L/mg
	R^2^ = 0.9947	R^2^ = 0.9921	R^2^ = 0.9829	R^2^ = 0.9931
Sep	q_max_ = 6.8728 mg/g	K_F_ = 0.2419 (mg/g)(L/mg)^1/n^	A_T_ = 2.2714 L/mg	β_S_ = 0.8179
	K_L_ = 0.0274 L/mg	1/n = 0.7718	b_T_ = 2.2856 kJ/mol	K_S_ = 0.0032 L/mg
	R^2^ = 0.897	R^2^ = 0.9952	R^2^ = 0.9391	R^2^ = 0.9951
AgNPs@Sep	q_max_ = 5.9347 mg/g	K_F_ = 1.8061 (mg/g)(L/mg)^1/n^	A_T_ = 1.3171 L/mg	β_S_ = 0.6667
	K_L_ = 0.0740 L/mg	1/n = 0.6376	b_T_ = 1.9481 kJ/mol	K_S_ = 0.0013 L/mg
	R^2^ = 0.9978	R^2^ = 0.9891	R^2^ = 0.9889	R^2^ = 0.9908
Ben	q_max_ = 4.6860 mg/g	K_F_ = 5.8761 (mg/g)(L/mg)^1/n^	A_T_ = 2.350 L/mg	β_S_ = 0.8028
	K_L_ = 0.0290 L/mg	1/n = 0.7760	b_T_ = 3.1373 kJ/mol	K_S_ = 0.0015 L/mg
	R^2^ = 0.9777	R^2^ = 0.9903	R^2^ = 0.9784	R^2^ = 0.9917
AgNPs@Ben	q_max_ = 6.0132 mg/g	K_F_ = 1.9201 (mg/g)(L/mg)^1/n^	A_T_ = 1.3410 L/mg	β_S_ = 0.6931
	K_L_ = 0.0702 L/mg	1/n = 0.6572	b_T_ = 1.9576 kJ/mol	K_S_ = 0.0020 L/mg
	R^2^ = 0.9945	R^2^ = 0.9821	R^2^ = 0.9907	R^2^ = 0.9849
DE	q_max_ = 3.2605 mg/g	K_F_ = 1.6449 (mg/g)(L/mg)^1/n^	A_T_ = 1.2740 L/mg	β_S_ = 0.4816
	K_L_ = 1.340 L/mg	1/n = 0.459	b_T_ = 3.4301 kJ/mol	K_S_ = 0.0002 L/mg
	R^2^ = 0.9754	R^2^ = 0.9688	R^2^ = 0.9744	R^2^ = 0.9704
AgNPs@DE	q_max_ = 5.5218 mg/g	K_F_ = 1.1014 (mg/g)(L/mg)^1/n^	A_T_ = 1.3008 L/mg	β_S_ = 0.533
	K_L_ = 0.1338 L/mg	1/n = 0.5184	b_T_ = 2.0316 kJ/mol	K_S_ = 0.0002 L/mg
	R^2^ = 0.9871	R^2^ = 0.9876	R^2^ = 0.9836	R^2^ = 0.9884

**Table 5:** Specific Surface Area of raw and AgNPs-functionalized silicate materials.

Silicate Material	Raw Material SSA (m^2^/g)	AgNPs-Decorated SSA (m^2^/g)	Key Structural Features	Refs.
Hal	57.3	14.27	Hollow nanotubes (lumens)	[45,51]
Sep	50–130	94.48	Interstratified layers	[47,52]
Ben	39–77	112	Fibrous bundles and channels	[46,53]
DE	17.4–27.8	39.0	Open macro-porous frustules	[48,50]

## Data Availability

The raw analytical data derived from the material characterizations, along with supplementary unpublished datasets, have been deposited in Zenodo and can be accessed via the DOI: https://doi.org/10.5281/zenodo.20712066.

## Supplementary Materials

The following are available online at https://www.mdpi.com/article/10.3390/ma19153268/s1, Figure S1: UV-Vis absorption spectra of the supernatants collected during the sequential wash-ing/centrifugation cycles (Washes 1, 2, and 3) of the four raw mineral substrates (Bentonite, Hal-loysite, Sepiolite, and Diatomite) following functionalization with silver nanoparticles. The com-plete disappearance of the characteristic LSPR band at 400 nm in the final cycles confirms the total immobilization of the nanoparticles onto the silicate matrices and the absence of residual, non-bound colloidal fractions in the waste solutions.
