# Supplementary material for: High-Efficiency Capture of Indole-3-Acetic Acid (IAA) from Water Using AgNPs-Decorated Silicate-Based Nanocomposites
Source: Materials (Basel). 2026 Aug 2;19(15):3268. doi: 10.3390/ma19153268 (PMC13467797; doi:10.3390/ma19153268)
Supplement: Supplementary file 1 [file materials-19-03268-s001.zip › materials-4431958-supplementary.pdf]

High-Efficiency Capture of Indole-3-Acetic Acid (IAA) from Water Using AgNPs-Decorated Silicate-Based Nanocomposites

Rosalia Maria Cigala [1,*,†], Ileana Ielo [1,*,†], Domenico Pio Basile [1], Francesco Paolo Lamonica [2], Paola Lanzafame [1], Georgia Papanikolaou [1], Giuseppe Zaffino [2], Francesco Crea [1,‡] and Giovanna De Luca [1,3,‡]

1 Department of Chemical, Biological, Pharmaceutical and Environmental Sciences, University of Messina, Viale F. Stagno d'Alcontres, 31-98166 Messina, Italy; domenico.basile@studenti.unime.it (D.P.B.); paola.lanzafame@unime.it (P.L.); georgia.papanikolaou@unime.it (G.P.); francesco.crea@unime.it (F.C.); giovanna.deluca@unime.it (G.D.L.)

2 Ambiente Lab—Technical Services for the Environment and Laboratory Chemical, Biological, Physical, and Environmental Analysis, Nuova Granatari 4 98164 Messina, Italy; francescopaolo.lamonica@gmail.com (F.P.L.); gzaffino@me.com (G.Z.)

3 National Research Council-Institute for Microelectronics and Microsystems (CNR-IMM), Viale F. Stagno d'Alcontres 31, 98158 Messina, Italy

* Correspondence: rosaliamaria.cigala@unime.it (R.M.C.); ileana.ielo1@unime.it (I.I.)

† These authors contributed equally to this work.

‡ These authors also contributed equally to this work.

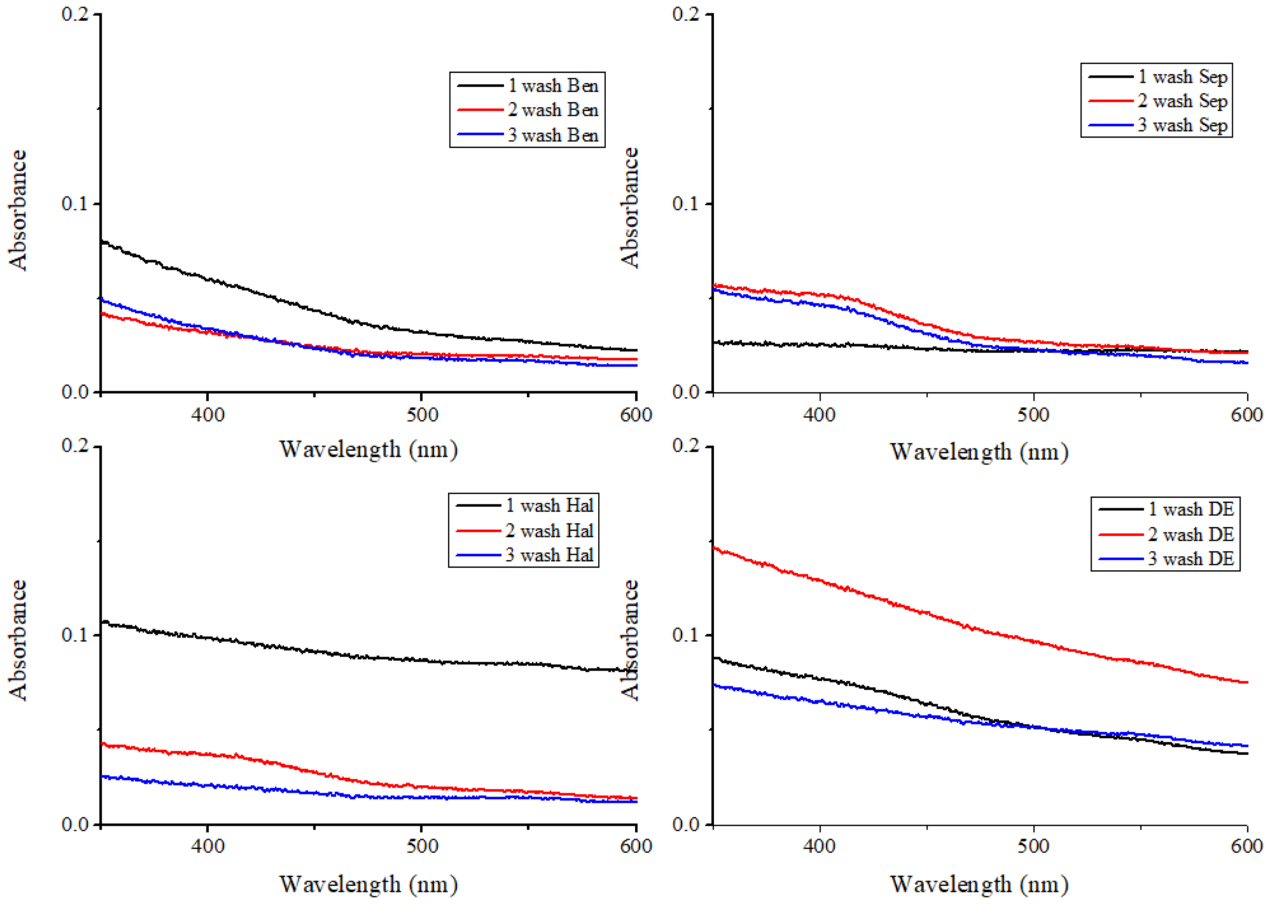

Figure S1 UV-Vis absorption spectra of the supernatants collected during the sequential washing/centrifugation cycles (Washes 1, 2, and 3) of the four raw mineral substrates (Bentonite, Halloysite, Sepiolite, and Diatomite) following functionalization with silver nanoparticles. The complete disappearance of the characteristic LSPR band at 400 nm in the final cycles confirms the total immobilization of the nanoparticles onto the silicate matrices and the absence of residual, non-bound colloidal fractions in the waste solutions.
